# Identification of BST-2/tetherin-induced hepatitis B virus restriction and hepatocyte-specific BST-2 inactivation

**DOI:** 10.1038/srep11736

**Published:** 2015-06-29

**Authors:** Mingyu Lv, Biao Zhang, Ying Shi, Zhu Han, Yan Zhang, Yulai Zhou, Wenyan Zhang, Junqi Niu, Xiao-Fang Yu

**Affiliations:** 1 Institute of Virology and AIDS Research, First Hospital of Jilin University, Changchun, P. R. China; 2 Department of Hepatology, First Hospital of Jilin University, Changchun, P. R. China; 3 School of Pharmaceutical Sciences, Jilin University, Changchun, P. R. China; 4 School of Life Sciences, Jilin University, Changchun, P. R. China; 5 Department of Molecular Microbiology and Immunology, Johns Hopkins Bloomberg School of Public Health, Baltimore, Maryland, USA

## Abstract

BST-2/tetherin is an interferon-inducible antiviral protein that blocks the release of various enveloped viruses, including HIV-1. Hepatitis B virus (HBV), a major cause of liver disease, belongs to the Hepadnaviridae family of enveloped DNA viruses. Whether BST-2 regulates HBV production is largely unknown. In this report, we have demonstrated that HBV particle release is modulated by BST-2 in a cell type-dependent fashion. In HEK293T cells, ectopically expressed or interferon-induced BST-2 strongly inhibited HBV release. BST-2 co-localized with HBV surface protein at multivesicular bodies (MVBs) and physically interacted with HBV particles. However, exogenous BST-2-induced HBV restriction was weak in Huh-7 hepatoma cells, and the interferon-induced anti-HBV effect was independent of BST-2 induction in hepatic L02 cells. Notably, HBV could promote HIV-1 ΔVpu virus release from BST-2-positive HepG2 hepatoma cells but not HeLa cells, whereas Vpu failed to efficiently inhibit BST-2-induced HBV restriction. HBx exhibited an enhanced interaction and co-localization with BST-2 in hepatocytes. These observations indicate that BST-2 restricts HBV production at intracellular MVBs but is inactivated by HBV through a novel mechanism requiring hepatocyte-specific cellular co-factors or a hepatocyte-specific environment. Further understanding of BST-2-induced HBV restriction may provide new therapeutic targets for future HBV treatments.

Viral hepatitis B is a major infectious disease initiated by the hepatitis B virus (HBV)^1^. HBV causes acute and chronic infection of the human liver and liver inflammation, giving rise to cirrhosis, and is a major risk factor for hepatocellular carcinoma (HCC)^2^. HBV is the prototype member of the Hepadnaviridae, which constitutes a small group of enveloped DNA viruses replicating via reverse transcription in hepatocytes of their specific host^3^. The HBV genome encodes the capsid protein (HBcAg), the envelope protein (HBsAg), a multifunctional protein exhibiting reverse transcriptase activity (P), and a regulatory protein (X). HBV assembly begins with the formation of nucleocapsids by capsid proteins, which are further enclosed by cellular lipids and viral surface glycoproteins, the small (SHBs), middle (MHBs), and large (LHBs) envelope proteins^4^. HBV virions (Dane particles) and subviral particles (filaments and spheres) are the main viral particles produced during the replication of HBV^5^. Another kind of capsid protein with a secretion signal (pre-capsid) translocates into the endoplasmic reticulum (ER) lumen for proteolytic processing and is secreted as a soluble protein, the hepatitis B e antigen (HBeAg)^5^. Capsid envelopment strictly depends on LHBs, whereas SHBs are required but not sufficient, and MHBs are dispensable^3^.

SHBs are a major component of the HBV envelope, and expression of SHBs alone is sufficient to generate empty subviral particles that bud at the post-ER/pre-medial-Golgi membranes and leave the cell via the classic constitutive pathway of secretion^6^,^7^. Formation of HBV virus progeny is initiated at the endoplasmic reticulum (ER), and then virus particle–containing vesicles (VCVs) are generated^8^. Notably, HBV formation requires the cellular machinery that generates internal multivesicular bodies (MVBs)^9^. Certain cell lines deriving from hepatocellular carcinomas, such as HepG2 and Huh7, produce infectious virus after transfection of the proviral constructs^10^. As the only accessory protein of HBV, the HBV x protein (HBx) is a necessary viral regulator of HBV replication and HBV-related HCC, which is multifunctional and highlighted in various cellular processes, including counteracting p53^11^, NF-κB activation^12^, cytosolic Ca^2+^ influx^13^, apoptotic regulation^14^, and autophagic degradation^15^. Interferon-α (IFN-α) therapy significantly clears HBV. However, the molecular mechanism(s) involved in the activity of interferon-stimulated genes (ISGs) and host anti-HBV factors remain far from completely understood^16^.

BST-2/tetherin blocks the release of enveloped viruses, including HIV-1, at the plasma membrane (PM)^17^. It contains a cytoplasmic tail, a transmembrane region, an extracellular domain, and a glycosyl-phosphatidlyinositol (GPI) anchor^18^. This structure is tightly connected with its antiviral function, defined as a “physical tethering” mechanism^19^. BST-2 is constitutively expressed in HeLa, H9, Jurkat, and Molt4 cells, as well as primary T lymphocytes and macrophages, but is absent from 293T, HOS, and HT1080 cells. The type-I IFN induces BST-2 expression in most BST-2-negative cells^17^. BST-2 exhibits a broad antiviral activity against enveloped viruses such as HIV, as well as other retroviruses and Lassa, Marburg, and Ebola virus-like particles^20^,^21^,^22^. Recently, it has been shown that BST-2 restricts some hepatotropic viruses. Several studies have proposed that BST-2 moderately inhibits hepatitis C virus (HCV) production^23^,^24^,^25^, while other results have implied that the activity is limited^26^. BST-2 can also inhibit dengue virus release from hepatoma cells^27^. Recent breakthroughs have added a novel function to the “physical tethering” function: human BST-2 activates NF-κB in response to viral tethering, acting as an innate sensor of viral assembly^28^,^29^.

As mentioned above, BST-2 tethers the nascent virions at the PM. There seems to be no obvious relationship between BST-2 and HBV production, which occurs in intracellular vesicles. Interestingly, a recent study presented evidence that BST-2 functions in intracellular compartments in macrophages, where BST-2 traps HIV-1 at the lumen of the intracellular vesicles^30^. We were interested in discovering whether BST-2 could suppress HBV production. In the present study, we have shown that HBV release is modulated by BST-2 in a cell type-dependent fashion. BST-2 strongly inhibited HBV release from HEK293T cells, but its effect was relatively weak in Huh-7 and L02 cells. BST-2 co-localized with HBV surface protein in multivesicular bodies (MVBs) and physically interacted with HBV particles. We also provide evidence that HBV counteracts BST-2 in hepatocytes. In a BST-2-positive hepatoma cell line (HepG2), the expression of HBV or HBx enhanced HIV-1 ΔVpu release. This HBV-induced BST-2 counteractivity differed from that known to occur with HIV-1 Vpu, and it occurred in intracellular compartments in the absence of surface removal of BST-2. Although we provide limited details about HBV-induced BST-2 inactivation, we have observed an enhanced interaction and co-localization between HBx and BST-2 in L02 hepatocytes. These data suggest that BST-2 restricts HBV production at the intracellular MVBs but is inactivated by HBV through a novel mechanism that requires hepatocyte-specific cellular co-factors or a hepatocyte-specific environment. This report may increase our understanding of BST-2 and provide new therapeutic targets for future HBV treatments.

## Results

### BST-2 effectively restricts HBV surface antigen release in 293T cells

A previous study has reported that the level of BST-2 expression is moderate in human liver^31^. However, BST-2 expression profiles in commonly used hepatic cell lines have not been systematically evaluated. Our study began with a test of endogenous BST-2 in non-hepatic and hepatoma cell lines. BST-2 was detected by flow cytometry. As shown in Fig. S1, 293T and Huh-7 cells were BST-2-negative, while HeLa and HepG2 cells were BST-2-positive. IFN-α further enhanced the BST-2 expression in each cell line. The level of endogenous BST-2 in HepG2 cells was higher than that in HeLa cells. However, the IFN-stimulated BST-2 enhancement in HepG2 cells was limited when compared with HeLa cells, revealing that the BST-2 gene is fully activated in HepG2 cells. To further confirm these BST-2 profiles, we analyzed BST-2 activity against HIV-1 WT and ΔVpu. The released p24CA level of HIV-1 ΔVpu was much lower than that of HIV-1 WT in HeLa and HepG2 cells, but no such discrepancy was seen in 293T and Huh-7 cells (Fig. S2).

**Figure 1:**
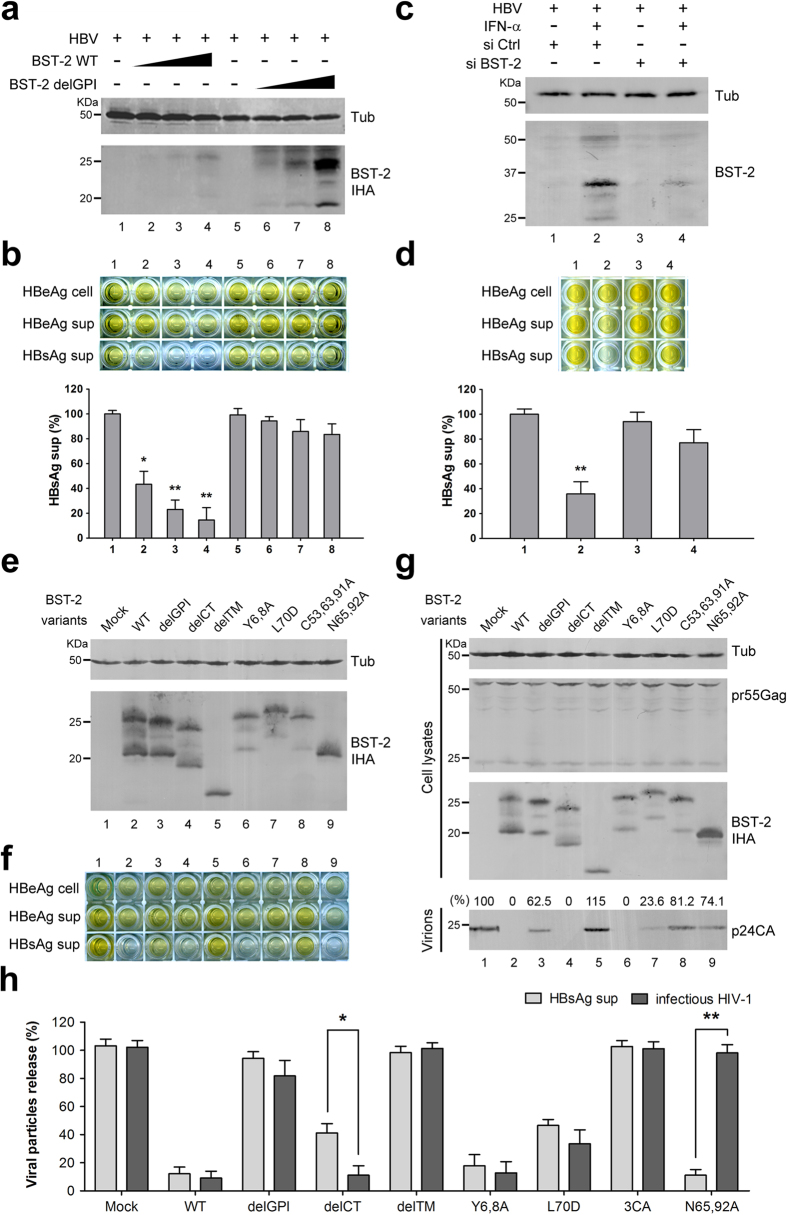
BST-2 effectively restricts HBV surface antigen release in 293T cells. (**a**) 293T cells were co-transfected with 0, 25, 50, or 100 ng of BST-2 IHA WT or delGPI plasmid along with 1 μg of HBV plasmid. BST-2 IHA was detected by Western blotting, and tubulin was used as a loading control. (**b**) HBV antigens in the cells and supernatants (sup) of a) were detected by ELISA. HBsAg release percentages are shown in columns. (**c**) 293T cells were transfected with 1 μg of HBV plasmid along with the control or BST-2 siRNA, followed by a treatment of 1000 U/ml IFN-α 24 h post-transfection. After another 48 h, BST-2 was detected with BST-2 antibody by Western blotting. (**d**) HBV antigen expression and release of c) were examined with ELISA, and HBsAg release percentages are shown in columns. (**e**) 293T cells were co-transfected with 1 μg of HBV construct, along with 50 ng of BST-2 variant. BST-2 IHA and tubulin were examined by Western blotting. (**f**) HBV antigen expression and release of e) were examined by ELISA. (**g**) 293T cells were co-transfected with 1 μg of pNL4-3 ΔVpu, along with 50 ng of BST-2 variant. BST-2 IHA, tubulin, cellular Gag, and released capsid were detected by Western blotting. (**h**) The HBsAg release in f) and the titration of the released infectious HIV-1 viruses in g) are shown with percentages. **P < 0.01, *P < 0.05. These experiments were performed no less than three times, all with similar results.

We then analyzed the effect of BST-2 expression on the release of HBsAg and HBeAg from 293T cells. 293T cells were transfected with increasing doses of BST-2 WT IHA plasmids and BST-2 delGPI IHA as a control. BST-2 was expressed in a dose-dependent manner (Fig. 1a), and BST-2 delGPI was expressed at a relatively higher level than BST-2 WT, consistent with previous studies^19^,^32^. The media were monitored by HBsAg/HBeAg ELISA, and cell lysates were monitored by HBeAg ELISA to normalize HBV expression. Cellular and released HBeAg were only slightly decreased by BST-2 (Fig. 1b). Whereas the released HBsAg was strikingly decreased in the presence of BST-2 (decreased to 10% at 100 ng BST-2 plasmid), HBsAg was barely affected by BST-2 delGPI. We then evaluated the anti-HBV activity of IFN-α-induced BST-2 in 293T cells: 1000 U/ml IFN-α apparently induced BST-2 expression (Fig. 1c), and the released HBsAg was decreased by more than 50%. This inhibition could be significantly diminished in the presence of the BST-2 siRNA, indicating a specific antiviral activity slightly weaker than that of the overexpressed BST-2 (Fig. 1d).

### Several BST-2 functional mutants are unable to restrict HBV

To better understand the characteristics of the BST-2-induced HBV restriction, we analyzed BST-2 mutants whose anti-HIV-1 activity had been studied previously. 293T cells were transfected with BST-2 variants, along with the HBV or HIV-1 plasmid. The expression and release of viral particles were examined (HBV: Fig. 1e,f; HIV-1: Fig. 1g,h). As shown in Fig. 1h, BST-2 delGPI and delTM were inactive against both HBV and HIV-1. BST-2 delCT has recently been demonstrated to inhibit HIV-1^33^, whereas here it exhibited attenuated activity against HBV. BST-2 Y6,8A was incapable of activating NF-κB^28^; however, it potently inhibited HBV and HIV-1. BST-2 L70D, which failed to form tetramers^34^, exhibited considerable activity against HBV and HIV-1. In contrast, BST-2 C53,63,91A, which failed to form dimers^19^, retained little activity against either HBV or HIV-1. The glycosylation mutant BST-2 N65,92A failed to inhibit HIV-1, a finding that is consistent with previous studies^19^; however, it strikingly restricted HBV.

### HBs co-localizes with BST-2 in a CD63^+^ intracellular compartment

**Figure 2:**
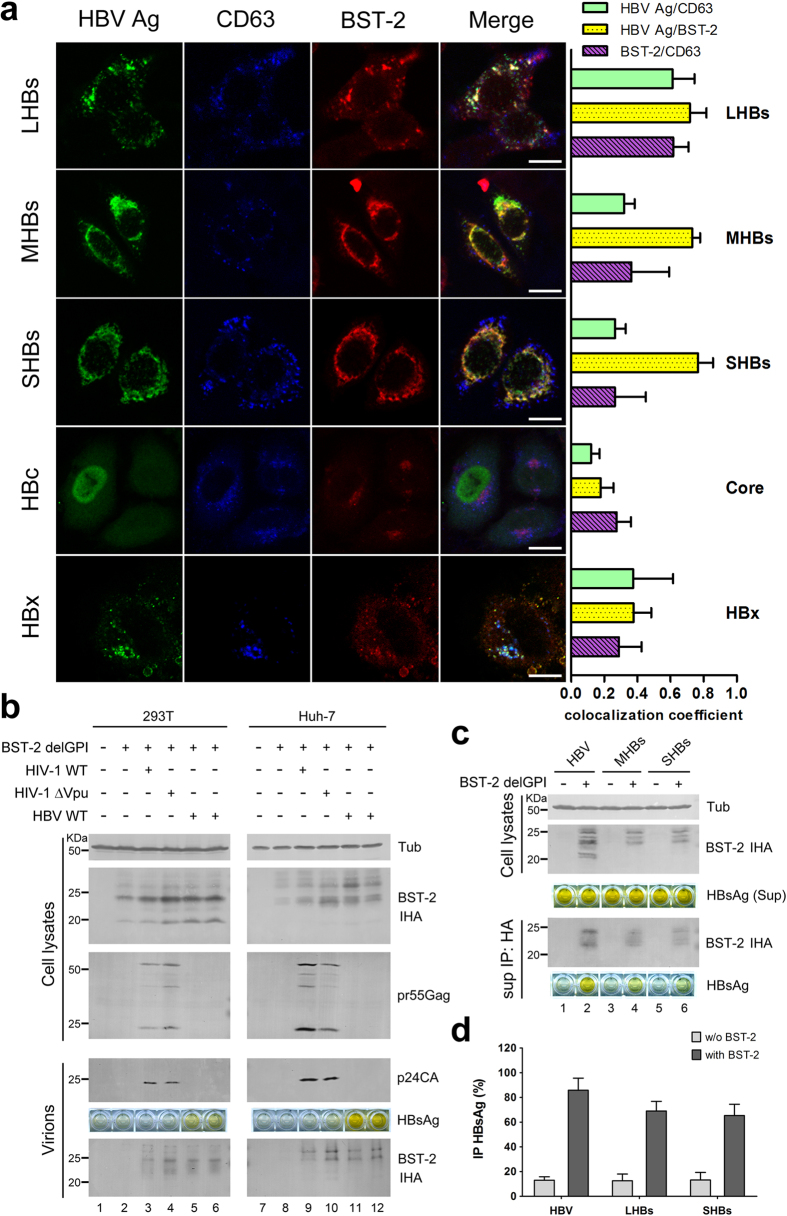
BST-2 co-localizes with HBs and physically interacts with HBV enveloped particles. (**a**) HeLa cells on coverslips, transfected with 500 ng of HBV protein plasmid and 100 ng of BST-2 IHA plasmid, were double-stained with a mouse anti-Flag antibody (mouse anti-myc for HBc) (secondary Ab: Alexa 488) and a rabbit anti-HA antibody (secondary Ab: Alexa 633), and further stained with a PE-conjugated anti-human CD63 antibody. Images were taken under a Zeiss LZM710 confocal microscope. Quantifications of the co-localization were performed using Image-Pro Plus 6.0. At least 20 independent images were examined in each sample, and the most representative cells are shown. Scale bars: 20 μm. (**b**) 293T and Huh-7 cells were co-transfected with 50 ng of BST-2 delGPI IHA and 1 μg of the indicated proviral plasmids. BST-2 and tubulin were examined by Western blotting. The virions were detected by Western blotting for p24CA and by ELISA for HBsAg. (**c**) 293T cells were co-transfected with 1 μg of HBV proviral, MHBs, or SHBs plasmid, respectively, with or without the BST-2 delGPI IHA plasmid. The clarified culture medium was examined by anti-HA immunoprecipitation. BST-2 was detected by Western blotting and HBsAg by ELISA. (**d**) The level of immunoprecipitated HBsAg in c) was quantified and shown in columns. These experiments were repeated three times, and the most representative data are shown.

HBV assembly occurs in intracellular vesicles that include MVBs. We next set out to define the co-localization of HBV proteins with BST-2, co-transfecting and fluorescently labeling both HBV and BST-2, then using these labeled molecules to determine the relationship of HBV proteins and BST-2 to MVBs. CD63 was labeled to indicate MVBs, as described in previous studies^35^. As shown in Fig. 2a, BST-2 exhibited a punctate distribution around the perinuclear region and also spread to the PM. HBs, and particularly LHBs, notably co-localized with BST-2 (71.6%). HBc exhibited a cytosolic distribution and barely co-localized with BST-2 (17.9%). HBx appeared as larger puncta; however, it exhibited limited but detectable co-localization with BST-2 (37.6%). HBc barely co-localized with CD63 (12.1%), and HBx slightly co-localized with CD63 (37.5%), whereas LHBs partially co-localized with CD63 (61.2%) (cyan in Merge). In contrast, MHBs and SHBs were localized to the perinuclear region and were ER-associated (32% and 26.5% co-localized with CD63). BST-2 partially co-localized with CD63 (purple in Merge), especially in some BST-2 puncta in the presence of LHBs (61.6%). Some bright yellow puncta near the PM in the LHBs image may represent sites at which the HBV assembly encounters BST-2 inhibition.

### BST-2 protein is incorporated into HBV enveloped particles

The association of BST-2 with viral particles depends on the interaction of the BST-2 membrane-spanning region with the lipid of virion envelope. To investigate whether BST-2 interacts with HBV particles according to this established mechanism, we co-transfected 293T cells with the BST-2 delGPI plasmid, along with either the HIV-1 or HBV proviral construct. Ultracentrifugation of the cultured supernatant was carried out to pellet the particles. As shown in Fig. 2b, little BST-2 was deposited in the absence of virus, but BST-2 became detectable in virion samples in the presence of HIV-1. BST-2 delGPI was detectable in the presence of HBV particles and was comparable to that pelleted with HIV-1. The association between BST-2 and HBV was also observed in Huh-7 cells (Fig. 2b, right panel).

The association of BST-2 and HBV-enveloped particles was further demonstrated by co-immunoprecipitation. HBV proviral construct, MHBs, and SHBs plasmids were respectively transfected with or without the BST-2 delGPI plasmid. Immunoprecipitation with HA affinity agarose beads was carried out on the cultured supernatants to collect the BST-2 delGPI protein. The HBsAg ELISA was positive when the BST-2 delGPI protein was efficiently immunoprecipitated, whereas the control sample without the BST-2 delGPI transfection was HBsAg-negative (Fig. 2c,d).

Studies have established that BST-2-induced NF-κB activation is stimulated during the antiviral process of BST-2, when it acts as a viral sensor of the immune system (reviewed in^36^). To further demonstrate the interaction between BST-2 and HBV particles, we tested the effect of HBV on BST-2-induced NF-κB activation. As shown in Fig. S3, BST-2-induced NF-κB signaling was enhanced by almost 10-fold in the presence of HBV expression.

### BST-2 restricts HBV less efficiently in Huh-7 hepatoma cells and L02 hepatocytes

**Figure 3:**
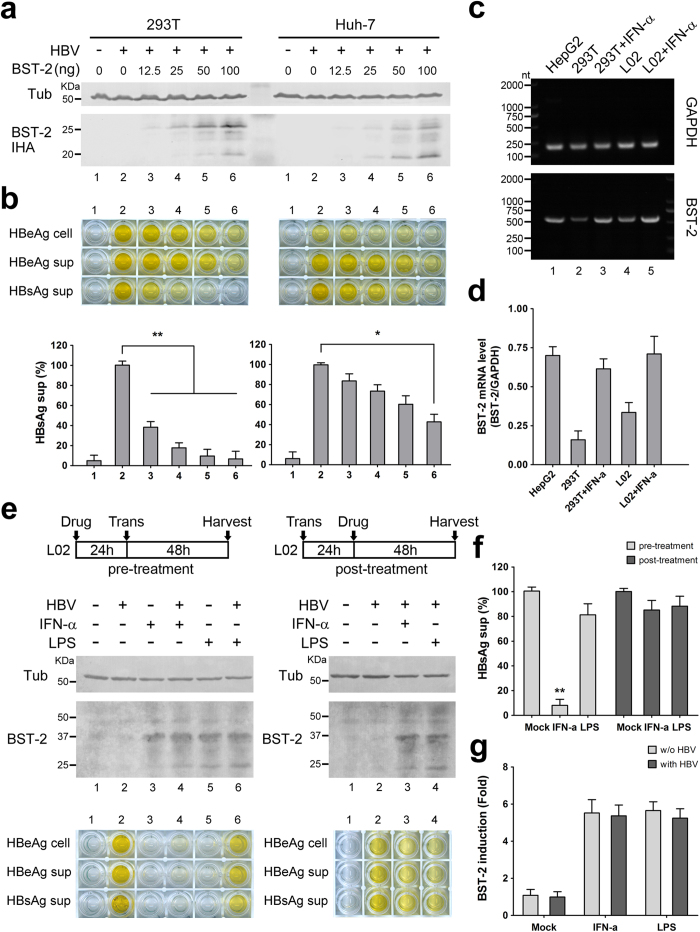
BST-2 restricts HBV less efficiently in hepatocytes. (**a**) 293T and Huh-7 cells were transfected with 0, 12.5, 25, 50, or 100 ng of BST-2 IHA and 1 μg of HBV proviral construct. BST-2 IHA and tubulin were detected by Western blotting. (**b**) HBV antigens expression and release in a) were examined with ELISA, and HBsAg release percentages are shown. (**c**) Endogenous BST-2 profiles in hepatic L02 cells were examined by RT-PCR of BST-2 mRNA and compared to 293T and HepG2 cells. GAPDH was set as a control. (**d**) The BST-2 mRNA level of c) was quantified and normalized by the GAPDH level. The bands of the agarose gel were quantified using Bandscan software. (**e**) L02 cells were pre-treated or post-treated with 1000 U/ml IFN-α or 1000 ng/ml LPS or left untreated as indicated, with 1 μg of empty vector or HBV proviral construct transfection. BST-2 and tubulin were detected by Western blotting. HBV antigen expression and release were examined by ELISA. (**f**) HBsAg release percentages of e) are shown. (**g**) The induction (-fold) of BST-2 by the pre-treated drugs was quantified by scanning the BST-2 blots and is shown. **P < 0.01, *P < 0.05. These experiments were performed no less than three times, with similar results.

BST-2 expression and function were further evaluated in Huh-7 cells, which express a very low level of BST-2 (Fig. S1), in parallel with 293T cells. The level of ectopically expressed BST-2 was similar in the Huh-7 and 293T cells (Fig. 3a). BST-2 reduced the release of HBsAg from 293T cells, but this function was impaired in Huh-7 cells (Fig. 3b). Next, we wondered whether the IFN-α antiviral treatment included BST-2-induced HBV restriction in hepatocytes. We focused on IFN-α- or lipopolysaccharide (LPS)-induced BST-2 production in hepatic L02 cells, which are derived from primary hepatocytes. By detecting BST-2 mRNA through RT-PCR, we determined that L02 cells express a moderate level of endogenous BST-2 and could be further stimulated significantly by IFN-α (Fig. 3c,d). When L02 cells were pre-treated with the stimulus for 24 h and then transfected, both IFN-α and LPS enhanced BST-2 expression (Fig. 3e). HBeAg expression and HBsAg release were not affected by LPS, but IFN-α did show inhibition (Fig. 3f). In another experiment, the cells were first transfected for 24 h and then treated with drugs for 48 h, using an assay similar to that used in the treatment of 293T cells shown in Fig. 1c,d. Under these conditions, BST-2 was still up-regulated by both IFN-α and LPS (Fig. 3e). In contrast, IFN-α hardly inhibited HBsAg release (Fig. 3f). Similar results were observed in Huh-7 cells (data not shown). In addition, no significant difference in BST-2 was seen in the presence or absence of HBV (Fig. 3g). These findings suggest that IFN-α-induced HBV inhibition in hepatic cells mainly functions in the early phase of HBV replication and not in late phases, by increasing BST-2 expression.

### HIV-1 Vpu fails to efficiently inhibit BST-2-induced HBV restriction

**Figure 4:**
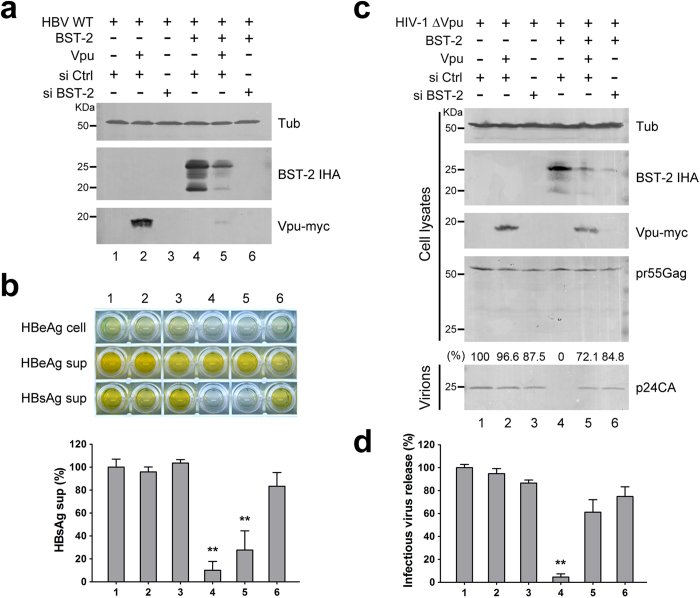
HIV-1 Vpu fails to efficiently inhibit BST-2-induced HBV restriction. (**a**) 293T cells were transfected with 1 μg of HBV proviral construct, 50 ng BST-2 IHA, 50 ng Vpu-myc plasmids, and siRNA as indicated. BST-2 IHA, Vpu-myc, and siRNA efficiency were detected by Western blotting. (**b**) HBV antigen expression and release in a) were examined with ELISA, and HBsAg release percentages are shown. (**c**) 293T cells were transfected with 1 μg of pNL4-3 ΔVpu proviral construct, 50 ng BST-2 IHA, 50 ng Vpu-myc plasmids, and siRNA as indicated. BST-2 IHA, Vpu-myc, siRNA efficiency, pr55Gag, and concentrated released p24 capsid were detected by Western blotting. (**d**) The titers of the released infectious HIV-1 viruses in c) were quantified and are shown in percentages. **P < 0.01. These experiments were performed three times, and the most representative Western blot is shown.

HIV-1 Vpu targets BST-2 via a transmembrane hydrophobic interaction and translocates BST-2 from the cell surface to the interior of the cell for degradation^37^,^38^. We wondered whether BST-2-induced HBV restriction could be inhibited by Vpu. As a control, BST-2 siRNA was applied to directly interfere with BST-2 expression. 293T cells were co-transfected with the HBV proviral construct, with or without BST-2 IHA plasmids. BST-2 and Vpu expression were examined by Western blotting (Fig. 4a). Neither Vpu expression nor BST-2 siRNA treatment had an observable effect on HBV expression and release (Fig. 4b, lanes 1–3). HBsAg release was notably decreased in the presence of BST-2 (Fig. 4b, lane 4). Although Vpu caused significant degradation of BST-2 (Fig. 4a, lane 5), BST-2-induced HBV restriction was not efficiently inhibited by Vpu (Fig. 4b, lane 5). Treatment with BST-2 siRNA decreased the BST-2 level (Fig. 4a, lane 6), and the impaired HBsAg release was largely restored (Fig. 4b, lane 6). In contrast, both Vpu expression and BST-2 interference led to efficient recovery of HIV-1 ΔVpu release (Fig. 4c,d).

### HBV expression enhances HIV-1 ΔVpu release in HepG2 hepatoma cells

**Figure 5:**
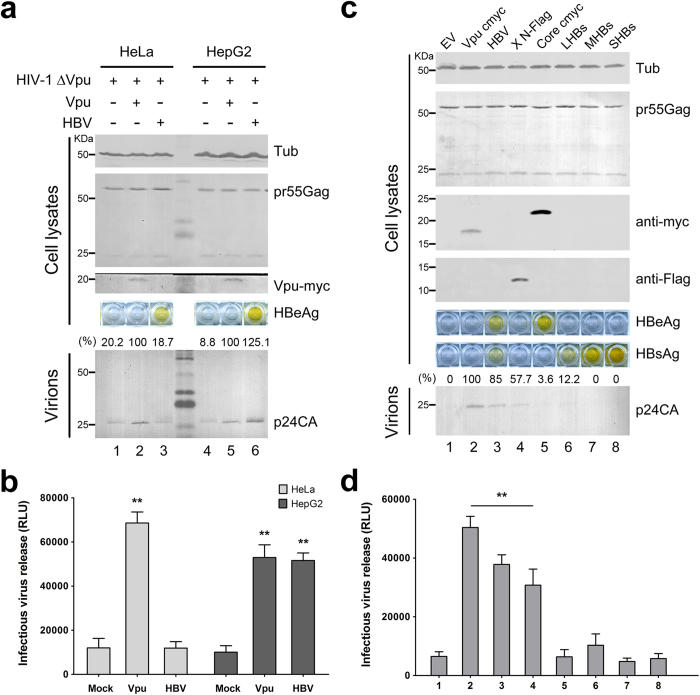
HBV expression enhances HIV-1 ΔVpu release in HepG2 cells. (**a**) HeLa and HepG2 cells were co-transfected with 1 μg of pNL4-3 ΔVpu proviral construct, along with 100 ng of empty vector, Vpu-myc vector, or HBV proviral construct. Vpu-myc, cellular pr55Gag, and concentrated released p24 capsid were detected by Western blotting. HBV antigen expression was determined by HBeAg ELISA. (**b**) The titers of the released infectious HIV-1 viruses in a) were quantified and are shown. (**c**) HepG2 cells were transfected with 1 μg of pNL4-3 ΔVpu proviral construct, along with 100 ng of empty vector, Vpu-myc vector, or HBV proviral or expression plasmid as indicated. Vpu and HBV core expression was examined by Western blotting with the anti-myc antibody, HBx with the anti-Flag antibody, and the cellular pr55Gag and concentrated release p24 capsid with anti-p24 antibody. HBs and HBe antigen expression was determined by HBsAg and HBeAg ELISA of cultured supernatants. (**d**) The titers of the released infectious HIV-1 viruses in c) were quantified and are shown. **P < 0.01. These experiments were repeated three times, and the most representative Western blot is shown.

The data presented above demonstrated that BST-2-induced HBV restriction in hepatoma and hepatic cells is weaker than that in 293T cells, indicating that HBV may counteract BST-2 in hepatocytes. To further confirm this possibility, we applied an indirect assay, evaluating the influence of HBV expression on HIV-1 ΔVpu release in BST-2-positive HeLa or HepG2 cells. In HeLa cells, Vpu expression efficiently enhanced HIV-1 ΔVpu release, but HBV expression had no such effect (Fig. 5a,b, lanes 1–3). In contrast, HBV expression considerably enhanced HIV-1 ΔVpu release, almost as potently as Vpu expression did in HepG2 cells (Fig. 5a,b, lanes 4–6). To further determine whether the enhancement was induced by one HBV protein or the whole virus, we individually expressed HBV Core, HBx, LHBs, MHBs, and SHBs to detect their effects on HIV-1 ΔVpu release in HepG2 cells. As shown in Fig. 5c, HBV Core, LHBs, MHBs, and SHBs had no significant influence on p24CA release, but HBx considerably enhanced HIV-1 ΔVpu release. The result was further confirmed by the infectivity assay (Fig. 5d). Consistent with the data shown in Fig. 3g, HBV and HBx did not dramatically down-regulate BST-2 level (data not shown).

### HBV accessory protein HBx induces a hepatocyte-specific accumulation of the intracellular BST-2

**Figure 6:**
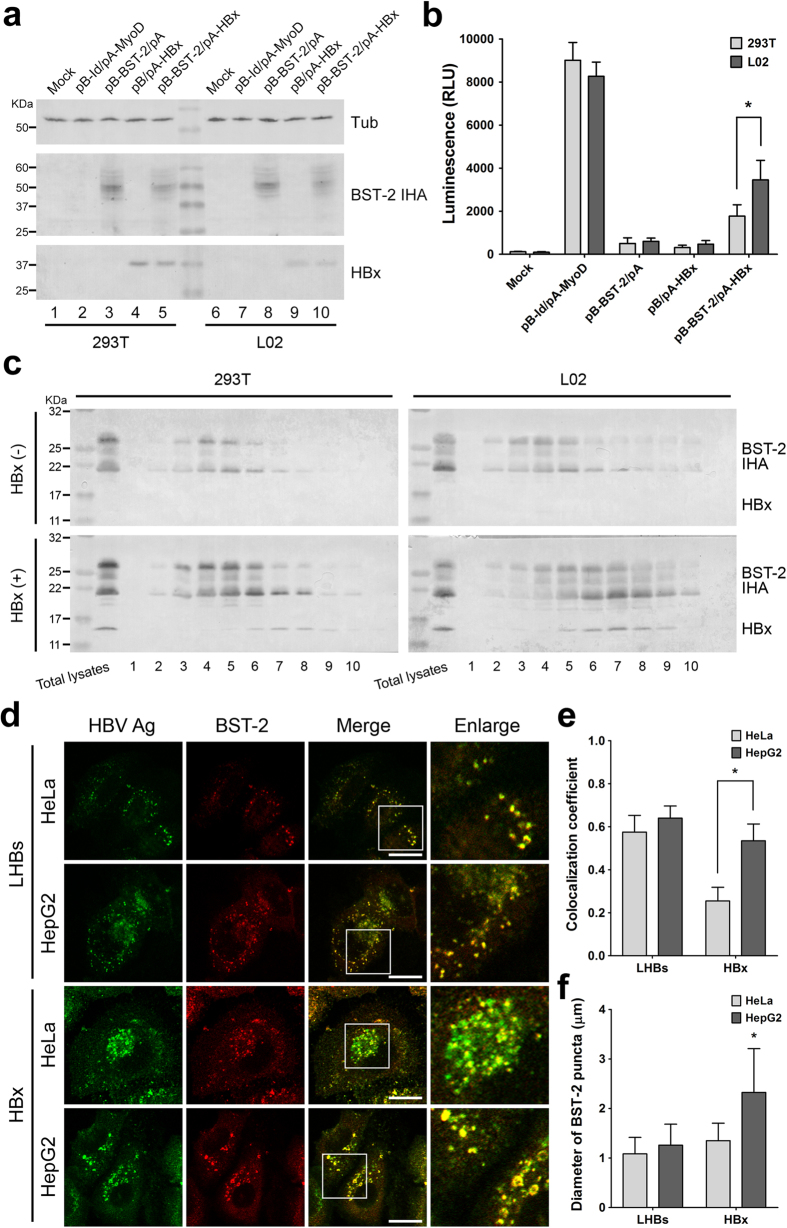
HBx induces a hepatocyte-specific BST-2 accumulation. (**a**) The interaction between BST-2 and HBx was analyzed by the protein two-hybrid assay in 293T and L02 cells. Cells were transfected with 500 ng of pBIND(pB)-BST-2 IHA and 500 ng of pACT(pA)-HBx, along with 500 ng of pG5luc vector as indicated. The expression of pBIND-BST-2 IHA and pACT-HBx was detected by Western blotting using anti-HA and anti-HBx antibodies. The pBIND-Id and pACT-MyoD pair is a commercial positive control. (**b**) Firefly luciferase values in a) were detected and normalized by *Renilla* luciferase values. (**c**) 293T and L02 cells in 10-cm dishes were transfected with 2 μg of BST-2 IHA plasmid in the presence or absence of 2 μg HBx plasmid. The cell lysates were separated by cellular fractionation. The samples were analyzed by Western blotting using a mixture of anti-HA and anti-HBx monoclonal antibodies to detect BST-2 and HBx on a same blot. (**d**) HeLa or HepG2 cells on coverslips, transfected with 500 ng of LHBs-Flag or Flag-HBx expression vector and 100 ng of BST-2 IHA plasmid, were fixed and double-stained with a mouse anti-Flag antibody (secondary Ab: Alexa 488) and a rabbit anti-HA antibody (secondary Ab: PE). Images were taken under a Zeiss LZM710 confocal microscope. Scale bars: 20 μm. (**e**) The co-localization coefficient of HBV protein and BST-2 in d) is shown. At least 20 independent images were examined in each sample, and the most representative cells are shown. (**f**) The diameters of the BST-2-localized cellular components in d) were measured with Image-Pro Plus 6.0 and are shown (n = 50). *P < 0.05. These experiments were performed three times, and the most representative data are shown.

The observations presented above indicated that the hepatocyte-specific BST-2 counteraction was induced, at least partially, by the HBV accessory protein HBx. We have attempted to provide more detailed information about the HBx-induced BST-2 counteractivity, but we have not detected a stable interaction of BST-2 and HBx via co-immunoprecipitation. However, we were able to apply a mammalian protein two-hybrid assay, in which BST-2 and HBx were respectively fused to a DNA-binding domain and a transcriptional activation domain of a transcription factor. This system produced a luciferase signal when the two proteins of interest came close enough together in the cells. The expression of the fusion proteins was confirmed (Fig. S4). Using this assay, we detected a closer association between BST-2 and HBx in hepatic L02 cells than in 293T cells (Fig. 6a,b). By fractionating the cell lysates through the gradient ultracentrifugation, we noticed that in the presence of HBx, BST-2 shifted into samples of larger density containing HBx, and this shift seemed to be more significant in hepatic L02 cells than in 293T cells (Fig. 6c). Consistent with this observation, we detected an enhanced co-localization of HBx and BST-2 in HepG2 cells when compared to HeLa cells, whereas the co-localization of LHBs and BST-2 was nearly the same in HeLa and HepG2 cells (Fig. 6d,e). More interestingly, in HepG2 cells, BST-2 was translocated into the larger puncta to which HBx was localized, and this phenomenon could not be observed in the presence of LHBs (Fig. 6f). All of these data point to a potential HBx/BST-2 interaction in hepatocytes that does not deplete the BST-2 level but does result in the capture and accumulation of BST-2, thus inactivating its antiviral function.

## Discussion

Defined as an antiviral protein against enveloped viruses, including HIV-1, BST-2 has recently been shown to be closely associated with hepatotropic viruses, including HCV^23^,^24^,^25^. HBV is another world-pandemic hepatotropic enveloped virus that shares distinct characteristics with HIV-1 and HCV. Significantly, HBV assembly occurs at the membranes of intracellular vesicles, including MVBs. Recent studies have presented evidence that BST-2 can modulate HIV-1 assembly in intracellular compartments in macrophages^30^. Hepatocytes undergo endocytosis and exocytosis similar to that of macrophages, indicating potential associations between BST-2 and HBV in these cells. Here we focused on BST-2 and HBV antigen release in hepatic/non-hepatic cell lines. We demonstrate that in HEK293T cells, both ectopically expressed and interferon-induced BST-2 suppressed the release of HBsAg (which represents the main HBV enveloped particles) but not HBeAg (the soluble viral protein secreting during HBV replication), consistent with already-known BST-2 behavior. Until now, most viruses defined as BST-2-sensitive have been found to be restricted to the PM. We found that the anti-HBV function of BST-2 is fairly dose-dependent (Fig. 1a,b), whereas IFN-α induced BST-2 activity to a moderate level when compared with overexpressed BST-2 (Fig. 1c,d), indicating that BST-2 inhibits intracellular particle assembly less efficiently than it does particle budding at the PM. Therefore, the anti-HBV function is more sensitive to the BST-2 level.

The BST-2-induced HBV restriction was further confirmed by evidence that demonstrated an interaction between BST-2 and HBV particles (Fig. 2b–d). Recycling endosomes, including MVBs, are potential BST-2 reservoirs during its intracellular trafficking and recycling from the cell surface^39^. BST-2 within the MVBs can be described as having its ectodomain in the lumen of the MVB and its cytodomain in the cytosol^18^,^19^. HBV particles assemble toward the lumen of the MVBs^40^, indicating that HBV encountering BST-2 at the MVB is equivalent to the antiviral model of BST-2 at the PM. HBV envelopment depends on the LHBs^5^. We found that BST-2 largely co-localized with the LHBs in MVBs (Fig. 2a), indicating that BST-2 recruits at the HBV assembly site. One group has recently found that BST-2 and the HCV core co-localize in lipid droplets (LDs), which constitute an important intracellular organelle for HCV assembly^25^. Our results suggest that BST-2-induced HBV restriction shares similarities with the anti-HCV model. As such, BST-2 N65,92A, which fails to traffic, may be enriched in intracellular vesicles, interfering with HBV but not HIV-1 (Fig. 1e–h).

IFN-α treatment is licensed for hepatitis B therapy and results in virus clearance in some patients^16^. However, its efficacy is limited by systemic side effects, and high doses are not tolerated^41^. In the present study, we have shown that IFN-α-induced BST-2 specifically inhibits HBV release from 293T cells (Fig. 1c,d). HBV expression was affected by IFN-α in hepatocytes (Fig. 3e), which may be related to the direct inhibition of HBV replication by IFN-α-induced proteins. For example, a very recent study has highlighted the finding that IFN-α can induce specific degradation of nuclear viral DNA without toxicity, through the up-regulation of APOBEC3A and 3B cytidine deaminases^42^. Considering these factors, we can account for the observation that BST-2 function in hepatic IFN-α treatment is modest, mainly because of the potential HBV neutralization. Another recent study has demonstrated that TLR-4 engagement with LPS enhances BST-2 activity, whereas IFN-α is less effective^43^. LPS induces the re-localization of BST-2 to CD81^+^ tetraspanin-enriched microdomains. While CD63^+^ MVBs could fuse with late lysosomes and convert them to CD81^+^ compartments^30^, it will be interesting to further investigate the effect of LPS on BST-2-induced HBV restriction.

BST-2-induced HBV restriction cannot be efficiently inhibited by HIV-1 Vpu expression (Fig. 4). To some extent, this observation may be explained by the widely accepted notion that Vpu effectively down-regulates BST-2 from the cell surface but only exhibits a modest effect on its total cellular level. HIV-1 Vpu effectively counteracts BST-2 activity against PM-budding particles, but counteracts BST-2 activity against intracellular particles less effectively. This idea is consistent with a recent report suggesting that BST-2 inhibits the release of HIV-1 particles from monocyte-derived macrophages, where HIV-1 exhibits a tendency to bud into intracellular vesicles, and that this function is relatively insensitive to HIV-1 Vpu^30^.

This anti-HBV activity is relatively higher in non-hepatocytes than in hepatocytes. These results suggest that HBV may overcome BST-2 restriction, which requires hepatocyte-specific factors or a hepatocyte-specific environment. Based on our observation that BST-2 inhibits HIV-1 ΔVpu in HepG2 and HeLa cells (Figs S2 and 5a), we found that HBV could promote HIV-1 ΔVpu virus release from BST-2-positive HepG2 hepatoma cells but not from non-hepatic HeLa cells. In addition, HBV potently enhanced BST-2-induced NF-κB activation (Fig. S3). Importantly, BST-2 co-localized with HBV surface protein (LHBs) at the MVBs and physically interacted with HBV particles (Fig. 2). All of these observations suggest a hepatocyte-specific HBV-mediated BST-2 counteractivity. The fact that HBV enhanced HIV-1 ΔVpu release in HepG2 cells but not in HeLa cells strongly indicates that HBV may counteract BST-2 in hepatic cells (Fig. 5a,b). Further experiments indicated that the viral antagonist may be the multifunctional accessory protein HBx, at least to some extent (Fig. 5c,d). The antagonism resulted in no detectable down-regulation of cell-surface BST-2. However, a close association of BST-2 and HBx was determined in hepatic L02 cells by the CheckMate™ Mammalian Two-Hybrid System (Fig. 6a,b), suggesting that a hepatocyte-specific interaction occurs between HBx and BST-2. Further, an HBx-induced cellular BST-2 translocation was exhibited in our cellular fractionation experiments, especially in L02 cells (Fig. 6c). In addition, an enhanced co-localization between HBx and BST-2 was observed in HepG2 cells (Fig. 6d,e). This evidence points to an HBx-induced inactivation of BST-2-restricted intracellular particle formation. This particular observation is especially similar to the Ebola virus situation. Ebola glycoprotein (GP) can substitute for Vpu to promote HIV-1 virion release from BST-2-expressing cells^22^ without the removal of the antiviral factor from the cell surface or lipid rafts^44^,^45^,^46^. Despite these observations, a more recent study has also demonstrated that in certain cell lines, even HIV-1 Vpu can stabilize BST-2 expression and counteract its antiviral activity^47^. Alternatively, the HBx-induced BST-2 inactivation may take place during the recycling of BST-2 from the cell surface, and this possibility may explain why HBx enhances HIV-1 ΔVpu release without down-regulating the antiviral factor.

**Figure 7:**
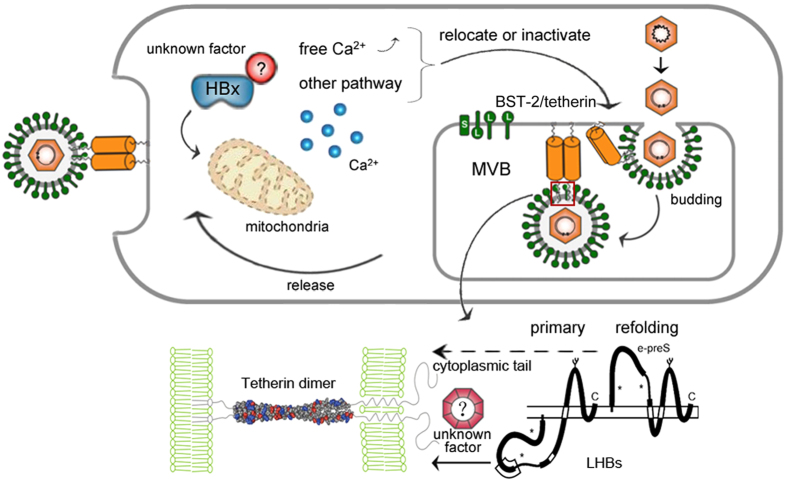
A schematic model of the HBV/BST-2 counteractivity. BST-2 tethers HBV at the lumen of MVBs, restricting HBV particle formation and release after the fusion of MVBs with the plasma membrane. HBx co-localizes and potentially counteracts intracellular BST-2 and inactivates its antiviral function specifically in hepatocytes through an undefined mechanism.

Further efforts should be made to define how HBx contributes to the dysfunction of BST-2. One promising direction is suggested by the observation that in hepatocytes, HBx activates cytosolic Ca^2+^-dependent phosphatidylinositol-specific phospholipase C (PI-PLC)^13^, a phospholipase that can remove the GPI anchor domain of BST-2 (Fig. 7). A recent study has reported that HBx inhibits autophagic degradation by impairing lysosomal maturation in hepatocytes^15^, which can significantly increase the number and volume of autophagosomes. The interesting observation that BST-2 accumulates in the larger puncta to which HBx localizes in HepG2 cells (Fig. 6f) may be an indication of potential autophagic regulation of BST-2 by HBx. Actually, HBx did not eliminate BST-2 activity as strongly as did HBV expression, meaning that other possibilities still exist. Alternatively, the antagonism may be a consequence of the coordination of the viral life cycle. HBV may utilize the developed metabolic system and vesicle trafficking as well as the strong secretion in hepatocytes to enhance HIV-1 release. As such, BST-2 could be an essential regulator of the HBV envelopment in hepatocytes. The feline immunodeficiency virus (FIV) envelope has been defined as an antagonist of BST-2 without a significant BST-2 down-regulation^48^. However, a recent study has indicated that FIV actually requires BST-2 for optimal release^49^. Ongoing efforts should be made to clarify these apparently antagonistic results and to resolve the counteractivity between BST-2 and HBV, thereby suggesting new therapeutic targets for the future HBV treatments.

## Methods

### Plasmids and reagents

BST-2 with an internal HA tag (BST-2 IHA) was cloned as previously described^19^. BST-2 mutants (delGPI [ΔC20], delCT [ΔN20], delTM [ΔN45], Y6,8A, L70D, C53,63,91A, and N65,92A) were cloned based on BST-2 IHA with site-direct mutagenesis. HIV-1 NL4-3 Vpu-myc was derived from pcDNA-Vphu; together with the HIV-1 proviral clone pNL4-3, pNL4-3 ΔVpu was a gift from NIH-ARRRP. The pCMV ayw HBV proviral construct was described previously^50^. LHBs-Flag, MHBs-Flag, SHBs-Flag, HBc-myc, Flag-HBx and HBx were PCR-amplified from pCMV ayw HBV and cloned into VR1012. BST-2 and control siRNA were purchased from RIBOBIO.

### Cells and transfections

HEK293T (CRL-11268), HeLa (CCL-2), Huh-7 (PTA-4583), and HepG2 (77400) cells were purchased from the ATCC and cultured in DMEM supplemented with 10% serum, at 37 °C/5% CO_2_. Human hepatic L02 cells was purchased from the cell bank of the Chinese Academy of Science and cultured in DMEM supplemented with 10% serum, at 37 °C/5% CO_2_. DNA transfections were performed using polyethylenimine (SIGMA), while siRNA transfections were performed with Lipofectamine 2000 (Life Technologies). Unless otherwise indicated, the cells were cultured in 6-well plates for experiments.

### Virion production assay

For the HIV-1 and HBV analysis, viral particles were produced in the indicated cells in a 6-well plate with 1 μg of each proviral construct and the indicated amounts of other plasmids. The cultured medium of the producer cells was clarified with a 0.22-μm filter, and viral particles were pelleted through a 20% sucrose layer at 100,000 g for 2 h (for HIV-1) or 380,000 g for 16 h (for HBV analysis). Viral particle pellets resuspended in 30 μl RIPA buffer, along with the corresponding cell lysates, were analyzed by Western blotting or HBV antigen ELISA. The blots of released HIV-1 p24CA were quantified using Bandscan software and normalized by tubulin levels. In single-cycle infectivity assays, 50 μl of the filtered supernatant was mixed with DEAE-dextran (Sigma) at a final concentration of 15 μg/ml and incubated with TZM-bl indicator cells in a 96-well plate. At 48 h post-infection, the cells were lysed, mixed with luciferase substrates, and assayed for luciferase activity using a fluorescence microplate reader to measure released virion yield.

### Western blotting

The cells were lysed with RIPA buffer, mixed with SDS sample buffer, and heated at 99 °C for 10 min. The samples were then subjected to standard SDS-PAGE and transferred to a nitrocellulose membrane and incubated with primary antibody: For BST-2 IHA: mouse anti-HA monoclonal antibody (mAb) (Covance). For endogenous BST-2: rabbit anti-BST-2 polyclonal antibody (pAb) (NIH-ARRRP/Abcam). For Vpu-myc: mouse anti-myc mAb (Millipore). For Pr55Gag and p24CA: mouse anti-p24 mAb (NIH-ARRRP). For Flag-HBx: mouse anti-flag mAb (Covance). For HBx: mouse anti-HBx mAb (Santa Cruz). For VP16: mouse anti-HSV VP16 mAb (Santa Cruz). For tubulin: mouse anti-tubulin mAb (Covance), set as a loading control. Alkaline phosphatase-conjugated goat anti-mouse and goat anti-rabbit IgG (Jackson Immunoresearch) were used as secondary antibodies.

### HBV antigen ELISA

The cultured medium and cell lysates were examined for HBV surface antigen (HBsAg) and HBV e antigen (HBeAg) with ELISA kits (Kehua, Shanghai). The samples (50 μl/well) were incubated in the 96-well microplates at 37 °C for 1 h, followed by addition of 50 μl horseradish peroxidase-conjugated primary antibodies for 30 min, 50 μl substrate for 10 min, and finally 50 μl termination buffer. The microplate was imaged with a scanner (Hewlett-Packard) and quantified by a microplate reader (BIO-RAD). HBsAg in supernatants was normalized to the HBeAg expression level and converted into percentages.

### Immunofluorescence

Cells (20–50% confluent) seeded onto coverslips in 6-well plates were transfected as indicated. At 24 h post-transfection, the cells were fixed with 2% formaldehyde and permeabilized with 0.25% Triton X-100, blocked in 10% serum, and incubated with mouse anti-Flag mAb (Millipore) and rabbit anti-HA pAb (Covance) diluted 1:1000 for 2 h. They were then stained with Alexa Fluor 488-conjugated goat anti-mouse IgG and Alexa Fluor 633-conjugated goat anti-rabbit IgG (Molecular Probes) diluted 1:1000 for 1 h and further incubated with PE-conjugated CD63 mAb (Molecular Probes) diluted 1:500 overnight. After washing, the coverslips were mounted with mounting medium (SIGMA). The images were acquired on a Zeiss LSM710 confocal microscope and adjusted with ZEN software (Zeiss). The co-localization was quantified by converting RGB images to gray scale images, and overlapped images were measured for co-localization coefficient (R) using Image-Pro Plus 6.0 (Media Cybernetics). The measurement for the diameter of BST-2 puncta was analyzed by the same software.

### Co-immunoprecipitation

For the co-immunoprecipitation of BST-2 delGPI IHA protein and the released HBV particles, 293T cells seeded in 6-well plates were transfected with the indicated plasmids. Immunoprecipitations were performed on 1 ml of pre-cleared culture supernatants by adding 30 μl of HA beads (Roche) and rocking at 4 °C for 6 h. After five washes with PBS, the beads were resuspended in 30 μl glycine HCl (pH 2.0) elution buffer. The eluted samples were then analyzed by Western blotting and HBV antigen ELISA.

### RNA extraction and RT-PCR

Total cellular RNA from counted cells was extracted with TRIzol reagent (Invitrogen) following the manufacturer’s protocol and was fractionated on a 1.5% agarose gel to ensure equal loading. RT-PCR reaction was performed on thermocycler with one-step PrimeScript RT enzyme mix (TAKARA) using the following primers. BST-2 (543 bp): forward 5′-ATGGCATCTACTTCGTATG-3′, reverse 5′-TCACTGCAGCAGAGCGC-3′; GAPDH (220 bp): forward 5′-GAAGGTGAAGGTCGGAGTC-3′, reverse 5′-GAAGATGGTGATGGGATTTC-3′). The bands of the agarose gel were quantified using Bandscan software.

### Protein two-hybrid assay

The basis of two-hybrid systems is the modular domains of transcription factors: The association of a DNA-binding domain and a transcriptional activation domain promotes the assembly of RNA polymerase II complexes at the TATA box and increases transcription. In the CheckMate™ Mammalian Two-Hybrid System (Promega), the close association of one protein fused to a pBIND vector (contains the yeast GAL4 DNA-binding domain and *Renilla* luciferase as a transfection marker) with another protein fused to a pACT vector (contains herpes simplex virus VP16 activation domain) will increase transcription of the firefly luciferase reporter gene. The pBIND-Id and pACT-MyoD vectors are provided as a positive control. Here, BST-2 and HBx were respectively cloned into pBIND and pACT vectors. Cells were co-transfected with these vectors and pG5luc vector (contains GAL4 binding sites upstream of a minimal TATA box and a firefly luciferase gene) as indicated. After 48 h, the cells were lysed, and the firefly luciferase and *Renilla* luciferase were respectively examined with the Dual-Luciferase Reporter Assay System (Promega) according to the manufacturers’ instructions. Samples were analyzed in a Multilabel Plate Reader (Perkinelmer, VICTOR X2).

### Cellular fractionation

Cells plated in 10-cm dishes were transfected with the indicated plasmids. After 48 h, the cells were mixed with 1% Triton X-100 PBS buffer and rocked gently on ice for 30 min. The whole-cell lysates were subjected to sucrose density gradient centrifugation for isolation. The sucrose layer was prepared in a 12-ml centrifuge tube containing 1 ml each of 20%, 30%, 40%, 50%, and 60% sucrose in PBS. The gradients were spun at 35,000 rpm in a Beckman SW41 swing-out rotor for 16 h at 4 °C. Ten 0.5-ml fractions were collected from the top of the gradient.

### Flow cytometry

The cells seeded into 6-well plates and treated with the indicated reagents or left untreated. The cells were washed with PBS and blocked with 10% serum for 10 min, stained with rabbit anti-BST-2 pAb (NIH-ARRRP) or rabbit anti-HA (Covance) as a control, followed by Alexa Fluor 633-conjugated goat anti-rabbit IgG (Molecular Probes, Life Technologies), and analyzed on a FACSCali-bur (BD Biosciences). The data were analyzed with FlowJo 7.6.2 (Tree Star).

### Luciferase assay for detecting NF-κB activity

293T cells plated in 24-well plates were transfected with 25 ng of BST-2 expression plasmid along with 250 ng of pNF-κB-Luc reporter plasmid (Agilent) and 50 ng of *Renilla* luciferase plasmid as a transfection marker. Two days later, the cells were lysed, and firefly luciferase and *Renilla* luciferase were examined with the Dual-Luciferase Reporter Assay System (Promega) according to the manufacturers’ instructions. Samples were analyzed in a Multilabel Plate Reader (Perkinelmer, VICTOR X2).

### Statistical analysis

All the statistical data are presented as means ± SEM. Statistical significance of the differences was determined using Student’s *t*-test. Differences were considered significant at values of P < 0.05.

## Additional Information

**How to cite this article**: Lv, M. *et al.* Identification of BST-2/tetherin-induced hepatitis B virus restriction and hepatocyte-specific BST-2 inactivation. *Sci. Rep.* **5**, 11736; doi: 10.1038/srep11736 (2015).

## Supplementary Material

Supplementary Information
