# Supplementary material for: Identification of BST-2/tetherin-induced hepatitis B virus restriction and hepatocyte-specific BST-2 inactivation
Source: Sci Rep. 2015 Jun 29;5:11736. doi: 10.1038/srep11736 (PMC4484258; doi:10.1038/srep11736)
Supplement: Supplementary Information [file srep11736-s1.pdf]

Supplementary Information

Identification of BST-2/tetherin-induced hepatitis B virus restriction and hepatocyte-specific BST-2 inactivation

Mingyu Lv, Biao Zhang, Ying Shi, Zhu Han, Yulai Zhou, Yan Zhang, Wenyan Zhang, Junqi Niu, and Xiao-Fang Yu*

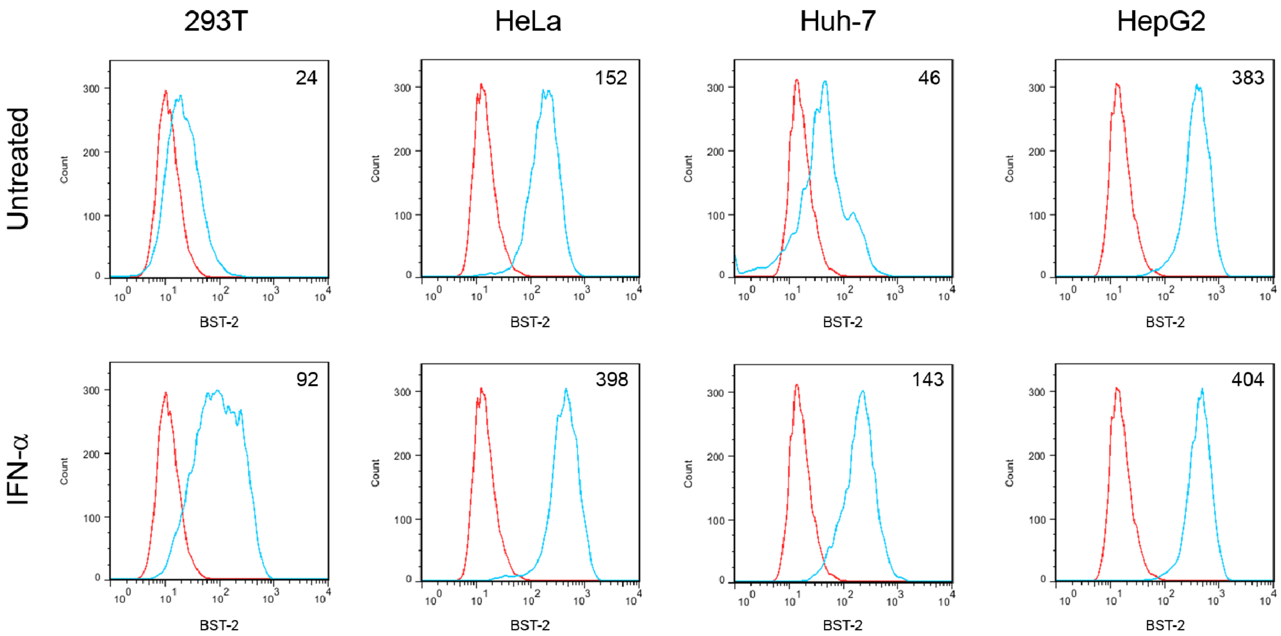

Figure S1. Profiles of endogenous BST-2 in hepatoma cells. 293T, HeLa, Huh-7, and HepG2 cells were untreated or treated with 1000 U/ml IFN-α and labeled with rabbit anti-BST-2 antibody and Alexa-633 goat anti-rabbit IgG, then counted by FACS. The surface BST-2 levels are shown in histograms with mean fluorescent intensitiy values at the top right corner. Red: control sample of each cell type stained with the control primary antibody (rabbit anti-HA). Blue: the test sample.

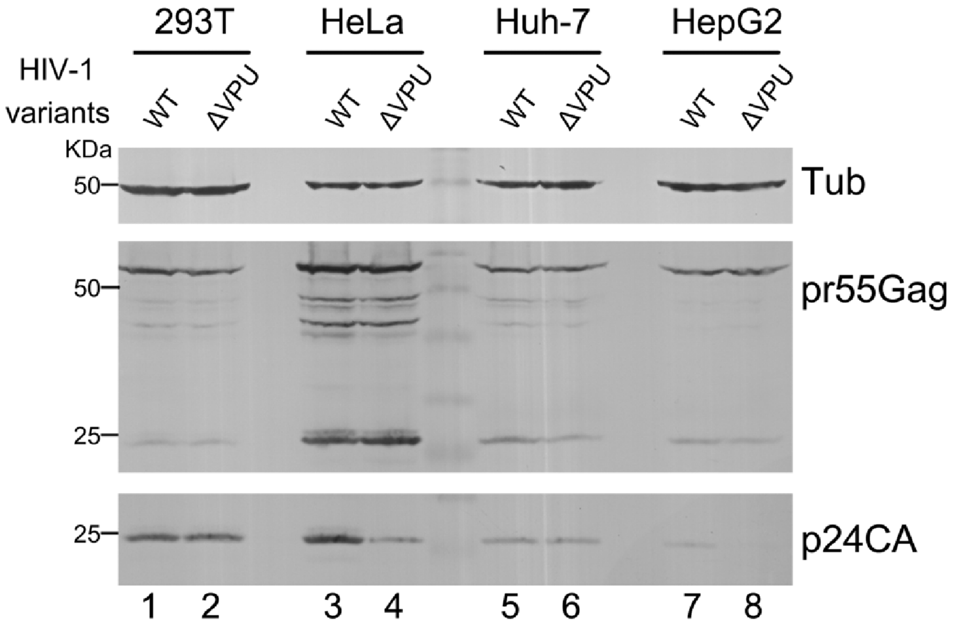

Figure S2. The analysis of anti-HIV-1 function of endogenous BST-2 in hepatoma cells. 293T, HeLa, Huh-7, HepG2 cells were respectively transfected with pNL4-3 WT or ΔVpu, the cells and virions were analyzed by Western blotting with p24 antibody.

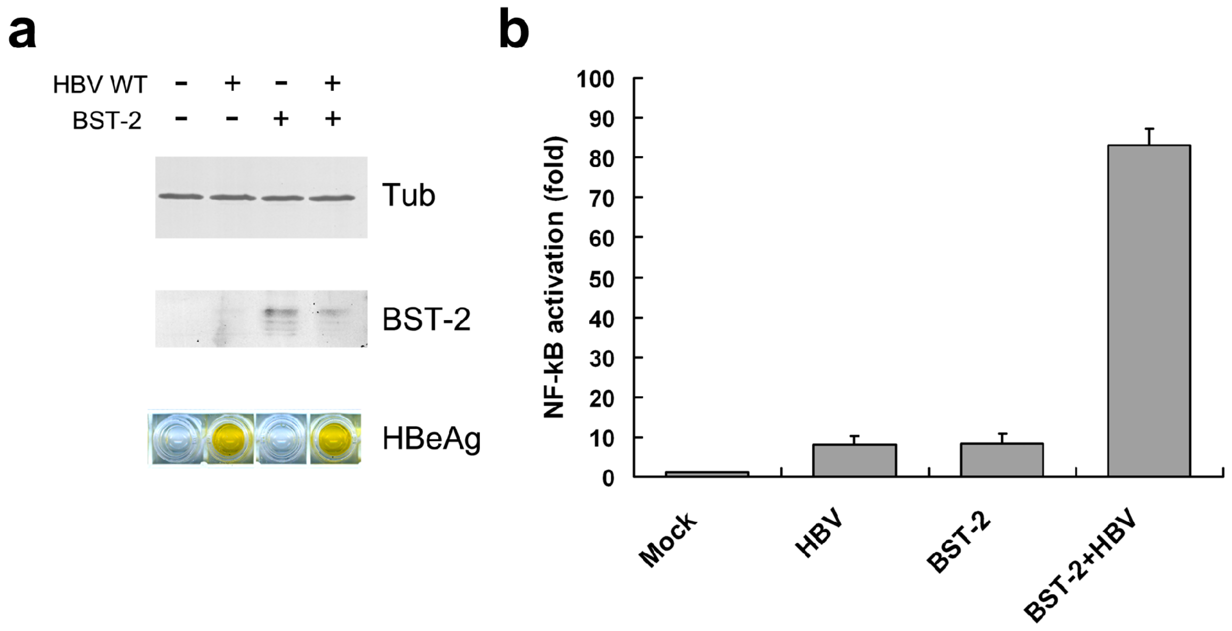

Figure S3. HBV potently enhances BST-2-induced NF-κB activation. (a) 293T cells were co-transfected with 25 ng BST-2 expression and 500 ng HBV plasmids as indicated, along with 250 ng of pNF-κB-Luc reporter plasmid and 50 ng Renilla luciferase plasmid. After 48 h, cells were lysed and analyzed for luciferase activity. BST-2 expression was analyzed by Western blotting. HBV expression was confirmed by ELISA. (b) The raw luciferase value was normalized to the renilla luciferase value. The data were compared with the mock control and shown as fold of NF-κB activation. The graph was generated from three independent experiments.

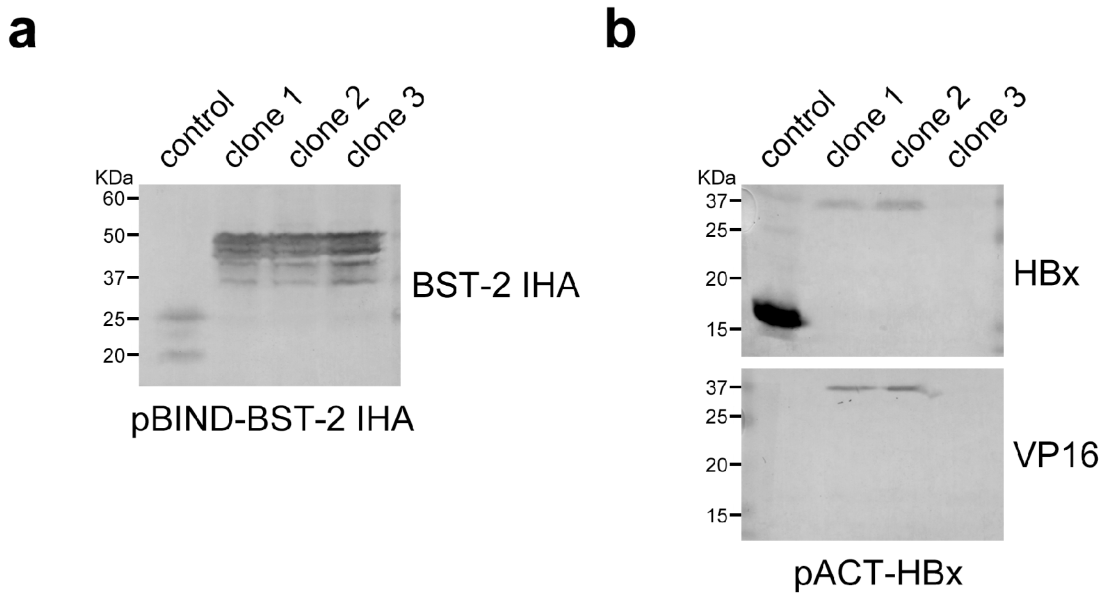

Figure S4. The expression test of the vectors for CheckMate™ Mammalian Two-Hybrid System. (a) 293T cells were transfected with 500 ng pBIND-BST-2 IHA plasmids (in which BST-2 IHA fused to yeast GAL4 DNA-binding domain) and BST-2 IHA VR1012 as the positive control. After 48 h, cells were analyzed for BST-2 expression by Western blotting using anti-HA antibody. (b) 293T cells were transfected with 500 ng pACT-HBx plasmids (in which HBx fused to herpes simplex virus VP16 activation domain) and HBx VR1012 as the positive control. After 48 h, cells were analyzed for HBx expression by Western blotting using anti-HBx antibody. Alternatively, an anti-VP16 antibody was also used to check the expression.
